# Pharmacogenetic testing in oncology: a Brazilian perspective

**DOI:** 10.6061/clinics/2018/e565s

**Published:** 2018-09-26

**Authors:** Guilherme Suarez-Kurtz

**Affiliations:** I Instituto Nacional de Cancer, Rio de Janeiro, RJ, BR; II Rede Nacional de Farmacogenetica, Rio de Janeiro, RJ, BR

**Keywords:** Pharmacogenes, Precision Medicine, Thiopurines, Fluoropyrimidines, Irinotecan, Tamoxifen, CYP2D6, DPYD, TPMT, UGT1A1

## Abstract

Pharmacogenetics, a major component of individualized or precision medicine, relies on human genetic diversity. The remarkable developments in sequencing technologies have revealed that the number of genetic variants modulating drug action is much higher than previously thought and that a true personalized prediction of drug response requires attention to rare mutations (minor allele frequency, MAF<1%) in addition to polymorphisms (MAF>1%) in pharmacogenes. This has major implications for the conceptual development and clinical implementation of pharmacogenetics. Drugs used in cancer treatment have been major targets of pharmacogenetics studies, encompassing both germline polymorphisms and somatic variants in the tumor genome. The present overview, however, has a narrower scope and is focused on germline cancer pharmacogenetics, more specifically, on drug/gene pairs for which pharmacogenetics-informed prescription guidelines have been published by the Clinical Pharmacogenetics Implementation Consortium and/or the Dutch Pharmacogenetic Working Group, namely, thiopurines/*TPMT*, fluoropyrimidines/*UGT1A1*, irinotecan/*UGT1A1* and tamoxifen/*CYP2D6*. I begin by reviewing the general principles of pharmacogenetics-informed prescription, pharmacogenetics testing and the perceived barriers to the adoption of routine pharmacogenetics testing in clinical practice. Then, I highlight aspects of the pharmacogenetics testing of the selected drug-gene pairs and finally present pharmacogenetics data from Brazilian studies pertinent to these drug-gene pairs. I conclude with the notion that pharmacogenetics testing has the potential to greatly benefit patients by enabling precision medicine applied to drug therapy, ensuring better efficacy and reducing the risk of adverse effects.

## INTRODUCTION

Pharmacogenetics (PGx), a major component of individualized or precision medicine, relies on human genetic diversity. The term pharmacogenetics was coined in 1959 1 to denote the study of the influence of genetic factors on the interindividual variability in drug response. The related term pharmacogenomics first appeared in the 1990s, in the wake of the “genomic revolution” brought about by genome-wide studies. The terms are often used interchangeably despite recognition that there are subtle differences, i.e., the effect of individual genes (pharmacogenetics) *versus* total genomic expression (pharmacogenomics), and I will use the abbreviation PGx to refer to both. The term pharmacogene will be applied to denote genes encoding proteins of importance for pharmacokinetics (drug absorption, distribution, metabolism and elimination) or pharmacodynamics (drug effects, whether beneficial or adverse).

As a result of the remarkable developments in next-generation sequencing technologies, human genomic variation has been characterized at an unprecedented level of detail, with major implications for the conceptual development and clinical implementation of PGx. It is now evident that the number of variants with importance for drug action is much higher than previously thought and that a true personalized prediction of drug response requires attention to rare mutations (minor allele frequency, MAF<1%) in addition to polymorphisms (MAF>1%) in pharmacogenes. Indeed, Kozyra et al. 2 identified a total of 12,152 exonic single-nucleotide variants in 146 pharmacogenes genotyped in over 6,600 individuals; most variants were rare (92.9%) or very rare (MAF<0.1%, 82.7%). These findings were confirmed and extended by Schärfe et al. 3, who detected 61,134 variants, predicted to be functional, in 806 pharmacogenes from over 60,000 exomes. The vast majority of these variants (97.5%) had an MAF<0.1%. Collectively, these data highlight the challenge to PGx implementation in clinical practice: a substantial effort will be required to catalog these variants and develop reliable algorithms to identify their putative functional effects and potential value as drug response biomarkers.

### PGx in oncology

**Figure 1:**
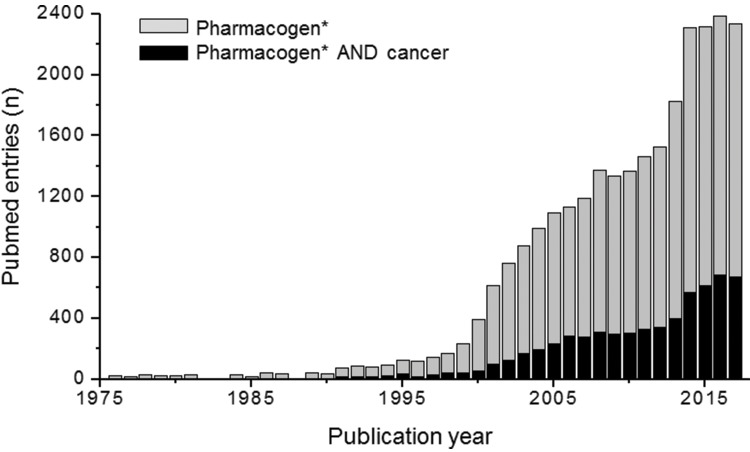
PubMed entries for the term “pharmacogen*” (gray columns) or “pharmacogen AND cancer” (https://www.ncbi.nlm.nih.gov/pubmed/ accessed January 5, 2018).

**Figure 2:**
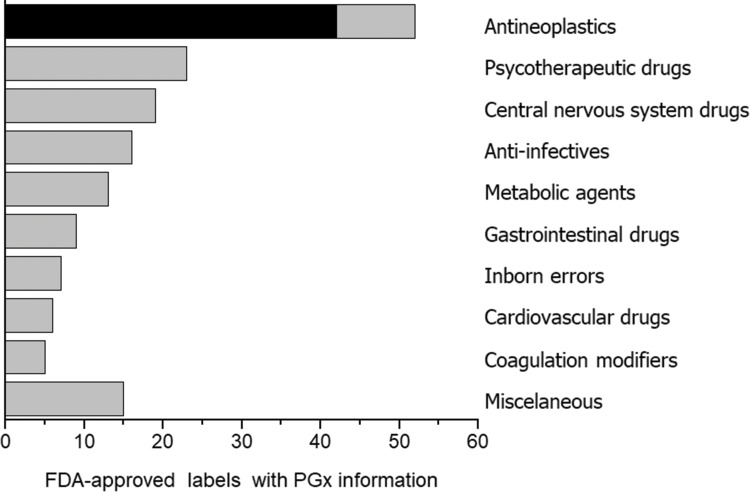
Number of FDA-approved labels with PGx information for the therapeutic classes listed on the right. Source: Table of pharmacogenetic biomarkers in drug labels, https://www.fda.gov/Drugs/ScienceResearch/ucm572698.htm, accessed Jan 5, 2018.

**Figure 3:**
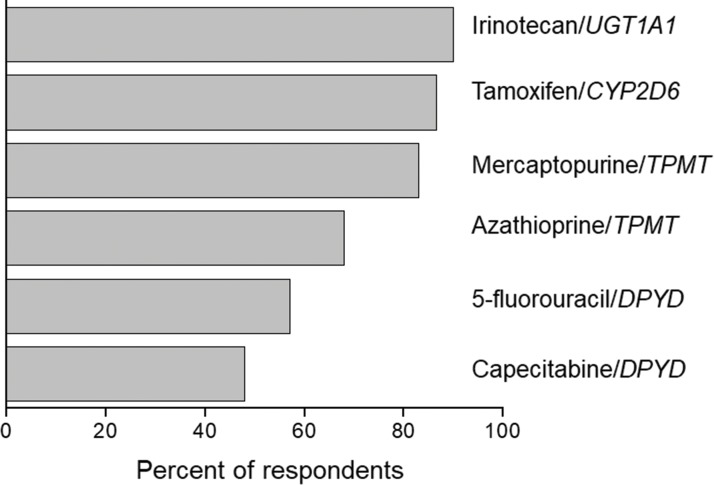
Data from a survey among United States physicians on their perception of the clinical relevance of PGx information. The bars correspond to the percentage of respondents who rated the antineoplastic/gene pairs listed on the right as 1 or 2 (on a scale of 1-5, where 1 is the most relevant). Source: Relling and Klein (78).

Among medical specialties, oncology has certainly been a major target for PGx studies and clinical implementation. This is reflected in the number of publications listed in PubMed for the combined terms PGx and oncology (Figure 1), in the proportion of drug labels approved by the United States Food and Drug Administration (FDA) that contain PGx information (Figure 2) and in the perception by physicians of the clinical importance of drug-gene interactions (Figure 3). While the latter survey covered only germline polymorphisms, both germline and somatic variants are represented in the PubMed data and in the FDA-approved drug labels. Indeed, the majority of PGx biomarkers in the labels for drugs used in oncology concern somatic mutations in tumor tissue (https://www.fda.gov/Drugs/ScienceResearch/ucm572698.htm). In addition to germline and somatic variants affecting pharmacokinetics or pharmacodynamics, PGx covers yet another area of interest to oncology, i.e., the activation and detoxification of carcinogenic xenobiotics by drug-metabolizing enzymes, such as CYP1A1, CYP2A6, GSTM1, and GSTT1, which are encoded by polymorphic genes. A broader scenario of the PGx of cancer would also comprise pharmacoepigenetics, i.e., heritable changes in the function of pharmacogenes that do not involve changes in the DNA sequence. These various facets of the PGx of cancer are covered in several excellent, recently published reviews 4-8. The present overview, however, has a narrower scope and is focused on germline cancer PGx, more specifically, on drug/gene pairs for which PGx-informed prescription guidelines have been published by the Clinical Pharmacogenetics Implementation Consortium (CPIC) and/or the Dutch Pharmacogenetic Working Group (DPWG). These gene-drug pairs are thiopurines/*TPMT*, fluoropyrimidines/*UGT1A1*, irinotecan/*UGT1A1* and tamoxifen/*CYP2D6.* I begin by reviewing the general principles of PGx-informed prescription, PGx testing and the perceived barriers to the adoption of routine PGx testing in clinical practice. Then, I highlight aspects of PGx testing of the selected drug-gene pairs and finally present PGx data from Brazilian studies pertinent to these drug-gene pairs.

### PGx-informed prescription

The goal of PGx is sometimes presented as providing the right dose of the right drug for the right patient, ideally at the onset of treatment. I suggest that a more realistic approach would be to view PGx as a valuable tool for informing drug prescription for the individual patient. In some cases, a PGx test may indeed provide decisive information for or against the prescription of a given drug. Oncologists are familiar with companion PGx testing for somatically acquired genetic variations in tumor tissue to guide the choice of anticancer drugs (e.g., imatinib, trastuzumab, and cetuximab). However, there are also germline variants that inform whether a drug may or may not be prescribed. A distinguished example is the increased risk of severe, life-threatening dermatological adverse reactions to carbamazepine in carriers of the HLA-B*1502 allele. This allele has a very distinct global distribution 9 with the highest frequency in populations of Asian descent but is absent or quite rare in African and European populations. Thus, the FDA recommends that “patients with ancestry in at-risk populations should be screened for the presence of the HLA-B*15:02 allele prior to starting carbamazepine,” whereas in Singapore, HLA-B*1502 testing has been adopted as the standard of care prior to the first use of carbamazepine 10.

Even when PGx tests are not required or recommended by regulatory agencies, they may still provide valuable information about drug efficacy or toxicity that is “actionable,” i.e., may be used to guide PGx-informed prescription. Guidelines for using actionable PGx information in clinical practice have been developed by the CPIC (htpps://cpicPGx.org/guidelines), the DPWG (https://www.pharmgkb.org/page/dwpg) and the Canadian Pharmacogenomics Network for Drug Safety (CPNDS, cpnds.ubc.ca). In total, over 70 dosing guidelines are currently available that take into account the patient's genetic profile (https://www.pharmgkb.org.view/dosing-guidelines.do). These guidelines rely on genotypic or (occasionally) phenotypic information that is already available and do not make recommendations for when, which or how PGx tests should be performed. Indeed, the timing and methodology of PGx tests for germline biomarkers is a matter of debate, specifically, whether the test(s) should be performed either reactively for targeted gene(s), with implications for a single drug at the time it is prescribed, or preemptively using a multigene panel, which provides genotype information for multiple pharmacogenes readily available in the patient's medical record to inform future drug therapy. Because germline PGx results have life-long validity, many consider preemptive genotyping a panel of PGx markers to be more relevant than genotyping for individual drug-gene pairs. However, this still requires systematic investigation.

### Implementation of PGx tests

The pros and cons of reactive *versus* preemptive PGx testing and the outcomes of PGx implementation using either approach across a variety of clinical settings were critically examined in recently published reviews 11,12. One aspect that I would like to emphasize is that optimization of the clinical utility of PGx tests, especially preemptive testing, depends not only on the accuracy of the genotyping procedures but also on the logistics of performing rapid turnaround genotyping and storing, interpreting, and making the PGx data readily available to the prescribing physician. The availability of and access to electronic medical record (EMR) systems are major factors for the successful implementation of PGx in clinical practice. Ideally, an EMR system would provide “friendly” access to the stored PGx information for authorized prescribing physicians not only from the institution where the PGx data were generated but also from other clinical settings where the patient may eventually be treated. In this regard, the lack of communication among currently available EMR systems represents a considerable caveat to the optimal use of preemptive testing. A comprehensive discussion of EMR PGx integration is available in a recent article by Caraballo et al. 13, based on their pioneer experience at the Mayo Clinic.

Other perceived barriers to the clinical implementation of PGx-informed prescription that require consideration are discussed below. Further insights into the barriers and solutions to the implementation of PGx testing may be found in a recently published review by Klein et al. 14.

### Paucity of clear clinical guidelines for translating genomic variations into actionable recommendations

The CPIC, DPWG and CPNDS guidelines (see above) contribute decisively to overcoming this barrier, but despite a high rate of concordance, differences among these guidelines do exist 15. A related factor is the disagreement among regulatory agencies with respect to requirement for and perceived clinical utility of PGx. This is illustrated in Table 1, with information regarding the chemotherapeutic drugs covered in the CPIC and/or DPWG guidelines, namely, mercaptopurines, fluoropyrimidines, irinotecan and tamoxifen. For example, DPWG, but not CPIC, published guidelines for irinotecan dosing according to UGT1A1 genotype, whereas among the four regulatory agencies listed in the table, only Health Canada/Santé Canada (HCSC) requires *CYP2D6* genotyping prior to the prescription of tamoxifen for breast cancer.

Discordance in recommendations for PGx testing extends to professional societies, a distinct example being fluoropyrimidines for colorectal cancer. Thus, CPIC and DPWG guidelines provide recommendations for drug prescription according to *DPYD* genotype and dihydropyrimidine dehydrogenase (DPD) activity, while the FDA and the Japanese regulatory agency, the PMDA, recognize *DPYD* information as actionable. However, the European Society of Medical Oncology (ESMO) consensus guidelines for the management of patients with metastatic colorectal cancer state that “DPD testing (....) remains an option but is not routinely recommended” and “none of the current strategies are adequate to mandate routine DPD testing” 16. Danesi et al. 17 argued strongly against these statements and pointed out that they “do not reflect the current awareness of the importance of testing for DPD deficiency …. (and) the Royal Dutch Pharmacists Association, the French GPCO-Unicancer group and the Italian Association of Medical Oncology-Italian Society of Pharmacology working groups have issued recommendations on preemptive DPD analysis for rational dose adaptation.” Further insights into the disagreements among regulatory bodies and professional societies regarding PGx may be found in a review by Gillis et al. 4.

### Paucity of prospective randomized clinical trials (RCTs) validating PGx-guided approaches

So far, prospective RCTs indicating the clinical utility of PGx tests to guide drug selection have been limited to carbamazepine 18, allopurinol 19 and abacavir 20. Nevertheless, many have argued that the scientific and clinical evidence supporting PGx clinical implementation is substantial for several other drugs (e.g., warfarin, clopidogrel, simvastatin, trastuzumab, and cetuximab), despite the lack of prospective RCTs 4,21,22. Pirmohamed and Hughes 21 proposed that the level of evidence required for the inclusion of PGx tests in treatment guidelines, drug labeling and reimbursement schemes should be equivalent to that required for nongenetic diagnostic tests. Altman 22 argued convincingly that the standard for adopting PGx-informed prescription should not be *superiority* to current practice but rather *noninferiority* (and the associated hypothesis of superiority). The validity of this evidence threshold is supported by the association between *TPMT* polymorphism and thiopurine toxicity, which never underwent an RCT and is yet the most validated and commonly used germline PGx test in clinical oncology.

### Confidence and knowledge of clinicians in accurately interpreting and acting upon PGx information

PGx is a relatively novel field, and current evidence points to a lack of preparedness among practicing clinicians to use PGx knowledge in routine practice. Indeed, in a survey covering over 10,000 US physicians, 97.6% agreed that genetic variations may influence drug response, but only 10.3% felt adequately informed about PGx testing, and only 29.0% reported receiving PGx education in either their graduate or postgraduate training 23. Accordingly, a survey of medical schools in the United States and Canada in 2010 revealed that only 28% provided more than 4 hours of instruction of PGx, and 76% considered that the provision of PGx instruction was poor or inadequate 24. Similar results were reported for British medical schools 25. Furthermore, PGx instruction, when provided, is included in the initial semesters of the medical curriculum, rather than later in the program when the students are more involved in patient care. PGx implementation, especially in a hospital setting, is not solely dependent on clinicians but also requires interaction with a multidisciplinary team including pharmacists, nurses and information technologists. Pharmacy schools, especially in North America, appear to be more active in implementing PGx education than medical schools 25,26. Pharmacists have been playing a key role in PGx adoption in clinical practice in the United States, and some predict that in the future, pharmacists will play a pivotal role in advising patients on individualized prescriptions 27.

### Cost and reimbursement aspects of PGx testing

As mentioned above, the availability of EMR systems are critical, if not indispensable, for the optimization of PGx-informed prescription. Computational tools for clinical decision support (CDS) will be required to prompt and guide clinicians to use genetic information when prescribing affected drugs. With the continuous decline in genotyping prices, the costs of PGx testing are shifting from laboratory testing toward the logistics of linking genetic test results to CDS systems that will robustly guide prescribing and will be routinely updated as new evidence emerges 28. Nevertheless, the frequency of PGx variants is a factor to be taken into account: for example, the combined frequency of the *TPMT* deleterious alleles listed in the CPIC guidelines for thiopurines is <5% among Brazilians (see below). Thus, on average, several hundred patients need to be genotyped to identify one carrier of two deleterious alleles who is at a 100% risk of severe myelotoxicity with conventional doses of mercaptopurine or azathioprine.

PGx testing has been examined is numerous pharmaco-economic analyses. A PubMed search with the terms “pharmacogen* AND cost-effectiveness” (pharmacogen* covers both pharmacogenetics and pharmacogenomics) yielded 178 review articles published in 2016 and 2017. The outcomes of pharmaco-economic analyses are not always concordant, which is not surprising considering the different methodologies applied, the country/region where the data were collected for the analyses, and the perspectives of the payer (e.g., patient, hospital, health insurance provider, and public health system). Exploring the reasons for this discordance is beyond the scope of this article, but I will briefly comment on selected results.

A systematic review of drug-induced adverse effects identified evidence supporting the cost-effectiveness of testing for HLA-B*57:01 (prior to abacavir), HLA-B*15:02 and HLA-A*31:01 (carbamazepine), HLA-B*58:01 (allopurinol), *CYP2C19* (clopidogrel) and *UGT1A1* (irinotecan); evidence was inconclusive for *TPMT* (thiopurines) 29. Verbelen et al. 30 carried out a thorough analysis of PGx testing for the biomarkers listed in the FDA-approved drug labels. Data for pharmaco-economic evaluation were available for 44 studies of 10 drugs, of which “57% drew conclusions in favor of PGx testing...30% were cost-effective (PGx was more effective at acceptable additional cost) and 27% were cost-saving/dominant (PGx was more effective at lower cost).”

### PGx testing in oncology

This section will briefly review the PGx tests for germline variants associated with cancer chemotherapy drugs, which have been included in the CPIC and/or DPWG guidelines and are listed in Table 1. Comprehensive information on the PGx of each drug/gene pair is available in the CPIC guidelines for thiopurines/*TPMT*, fluoropyrimidines/*DPYD* and tamoxifen/*CYP2D6* (https://cpicPGx.org/guidelines) and in recent reviews for irinotecan/*UGT1A1* 31,32.

### *TPMT* and thiopurines

Thiopurines (mercaptopurine (MP), thioguanine (TG), and azathioprine) are widely used anticancer and immunosuppressive agents. The three drugs share most pharmacological effects, but MP and azathioprine are commonly used for nonmalignant conditions (e.g., inflammatory bowel disease); additionally, MP is used for lymphoid malignancies, and TG is used for myeloid leukemias. Thiopurines are prodrugs, i.e., they must be converted into active thioguanine nucleotide (TGN) metabolites to exert their clinical benefits, as well as their adverse effects. Thiopurines are also substrates for other enzymes, which generate inactive metabolites; the major inactivating pathway is mediated by thiopurine methyltransferase (TPMT), encoded by the polymorphic gene *TPMT*. The opposing effects of the activating and inactivating enzymatic pathways determine the final concentrations of active TGNs, and, consequently, the magnitude of the effects of thiopurines. Several variant alleles of *TPMT* (e.g., *2, *3A, *3B, and *3C) encode nonfunctional TPMT isoforms, and there is substantial clinical evidence linking *TPMT* genotype to the phenotypic variability of TPMT. Individuals who inherit two inactive *TPMT* alleles are at a 100% risk for life-threatening myelosuppression if they are treated with conventional doses of thiopurines, whereas those who are heterozygous for nonfunctional alleles are at an increased risk, but only ∼30-60% appear to be unable to tolerate full doses of MP or azathioprine 33.

The CPIC *Thiopurine Methyltransferase Genotype and Thiopurine Dosing Guidelines* were first published in 2011 33, were updated in 2013 34 and will be updated again in 2018. These guidelines provide separate but similar recommendations for the three thiopurines, according to the individual *TPMT* genotype and inferred (or measured) TPMT activity: for a homozygous wild-type *TPMT* genotype or normal TMPT activity, start with the usual dose; for a heterozygous genotype or intermediate TMPT activity, consider starting with 30-70% of the target dose; for homozygous variants or markedly reduced TPMT activity, start with drastically reduced (10-fold) doses of MP or TG, and consider alternative agents if using azathioprine. All these recommendations, except for TG in individuals with a heterozygous *TPMT* genotype, are classified as “strong,” which is the highest of the three-level rating scale adopted by CPIC for evidence-based recommendations. For azathioprine in heterozygous individuals, the recommendation is rated “moderate.”

PGx testing for thiopurine/*TPMT* is routinely performed in several medical centers abroad for cancer and inflammatory bowel disease (IBD) patients. Relling et al. 35 reported the use of PGx-guided thiopurine therapy in acute lymphoblastic leukemia (ALL) since the early 1990s at the St. Jude Children's Hospital. A recent survey showed that six sites of the Translational Pharmacogenomics Program of the National Institutes of Health have cumulatively performed over 20,000 PGx tests for thiopurines/*TPMT*, with nearly 2,000 (9.4%) actionable results 12.

The results of cost-effectiveness analyses of *TPMT* PGx tests are inconsistent. For example, *TPMT* genotyping prior to thiopurine treatment in pediatric ALL was found to have a favorable cost-effectiveness ratio in Europe but not in the United Kingdom 36-37, whereas systematic analyses of PGx testing for thiopurines/*TPMT* revealed either inconclusive evidence 29 or cost-savings only when the data were analyzed assuming genotyping results were available at no extra cost 30. Nevertheless, it has been argued that “from an ethical point of view, it is highly questionable whether leukopenia/pancytopenia should be accepted in patients where screening for *TPMT* prior to thiopurine therapy can definitively identify TPMT deficiency, which leads in 100% of cases to hematotoxicity under standard dosage of thiopurines” 38. This view must be tempered with the notion that additional genetic and nongenetic factors may contribute to the toxicity of thiopurines, especially nonhematological adverse effects (e.g., pancreatitis and hepatotoxicity), which are poorly predicted by the *TPMT* genotypes for the *2 and *3A-*3C alleles. In addition to rare and/or not yet identified *TPMT* variants that may affect TPMT activity, there is evidence that polymorphisms in genes encoding other enzymes involved in thiopurine metabolism (e.g., *ITPA* and *NUDT15*) modulate systemic exposure to thiopurines. Indeed, the emerging role of *NUDT15* polymorphisms in thiopurine disposition and dose-related toxicity in ALL deserves special attention, particularly in patients of Asian descent 39.

### *DPYD* and fluoropyrimidines

The fluoropyrimidine 5-fluorouracil (5-FU) and its oral prodrug capecitabine (CAP) are commonly prescribed in the treatment of colorectal, stomach, breast and head and neck tumors. 5-FU has a narrow therapeutic index, and depending on the treatment regimen, up to 35% of patients suffer from severe, potentially fatal adverse effects, including myelosuppression, diarrhea, mucositis, hand-foot syndrome and neurotoxicity. The clinical implications of DPD deficiency in patients with severe 5-FU-associated toxicity were first reported in 2000 40. DPD, encoded by the polymorphic gene *DPYD*, is the rate-limiting enzyme in the inactivation of 5-FU in the human liver; consequently, reduced DPD activity leads to increased exposure to fluoropyrimidines.

Several variants in *DPYD* are known; of these, four associated with reduced DPD activity have been consistently associated with 5-FU toxicity. These variants are labeled DYPD*2A, DPYD*13, D949V (c2864A>T) and HapB3 (a haplotype of intronic SNPs, in complete linkage disequilibrium with the rs 7507182; Table 2). The first three variants are rare (MAF<1%) or very rare (MAF<0.1%), whereas HapB3 is relatively common, ranging in frequency from 4.1-4.8% (http://phase3browser.1000genomes.org/index.html). A meta-analysis of data from over 7,000 patients demonstrated that the risk of 5-FU-induced toxicity grade ≥3 is 1.6- to 4.4-fold greater in carriers of one or more defective *DYPD* alleles. The authors concluded that “upfront screening for these variants (DYPD*2A, *13, 2864T and HapB3) is recommended to improve the safety of patients with cancer treated with fluoropyrimidines” 41. Both CPIC and DWPC issued guidelines for the PGx-informed prescription of 5-FU and/or CAP (Table 2). The recently updated CPIC guidelines 42 adopted an activity score system to infer DPD phenotypes according to the genotypes at the 4 polymorphic sites listed above. Based on the inferred DPD phenotypes, the following recommendations are provided: 1 in poor DPD metabolizers (carriers of two deleterious *DPYD* alleles), avoid 5-FU and CAP; (II) in intermediate DPD metabolizers (carriers of one deleterious allele), reduce starting dose by 25-50% followed by titration of dose based on toxicity; (III) in normal DPD metabolizers (carriers of no deleterious alleles), use label-recommended dosage and administration 43. These CPIC recommendations were rated “strong,” except that for heterozygous carriers of reduced function alleles, which was rated “moderate.”

PGx tests for the variants listed in the CPIC guidelines consistently show high specificity but low sensitivity. For example, in a retrospective analysis of data from 603 Italian patients with colorectal cancer treated with 5-FU, both single and combination analyses of DPYD*2A, *13A and the 2846A>T SNP genotyping tests showed a specificity of 99-100%, while the sensitivity ranged from 1-12% 41. Low sensitivity as well as the low prevalence of *DPYD* functional variants are perceived as additional barriers to the adoption of PGx testing to prevent toxicity to fluoropyrimidines. Supporters of PGx testing argue that the stratification of patients on the basis of *DPYD* genotype may help prevent toxicity and is of the “utmost importance within a preventive, prognostic, and personalized approach to patient care in the oncology setting” 43; additionally, on a population level, upfront *DPYD* genotyping may save on costs 44. As alternatives to *DPYD* genotyping, several phenotypic methods have been described to assess, directly or indirectly, DPD enzymatic activity, but these methods are not included in the CPIC or DWPG guidelines and are not recommended by professional associations, such as the ESMO or the American Society of Clinical Oncology (ASCO).

### *UGT1A1* and irinotecan

Irinotecan is a topoisomerase-I inhibitor that is widely used in the treatment of metastatic colorectal cancer in combination with 5-FU/leucovorin (FOLFIRI) and bevacizumab. Irinotecan is a prodrug that requires *in vivo* conversion into the active metabolite 7-ethyl-10-hydroxycamptothecin (SN-38) to exert its pharmacological effects. SN-38 is eliminated predominantly by glucuronidation, in a reaction mediated primarily by UDP-glucuronosyltransferase 1A1 (UGT1A1), encoded by the *UGT1A1* gene. Systemic exposure to SN-38 is related to the number of TA base repeats in the promoter region of *UGT1A1.* The wild-type allele (UGT1A1*1) has six TA repeats, whereas a common variant allele (UGT1A1*28) has seven TA repeats. Other rarer variants at this locus are UGT1A1*36 (five repeats) and UGT1A1*37 (eight repeats). The gene transcription level is reduced by the seven and eight TA repeat alleles, and consequently, carriers of these alleles glucuronidate SN-38 less efficiently than patients with the wild-type genotype and are exposed to considerably higher plasma concentrations of SN-38. In patients of Asian descent, UGT1A1*6, an exonic SNP (c.211G>A, rs4148323), is the most common variant associated with reduced UGT1A1 metabolic activity 31,32. 

The association between UGT1A1*28 genotype and irinotecan toxicity, first reported in 2004 45, has been the subject of many studies and several meta-analyses, with conflicting results. For a comprehensive overview of this topic, which encompasses the UGT1A1*6 variant, the reader is referred to a recently published “umbrella” assessment of systematic reviews and meta-analyses 32; here, I will highlight the main conclusions of this umbrella analysis. The association of the UGT1A1*28 and *6 polymorphisms with an increased risk of developing irinotecan-induced neutropenia and diarrhea was confirmed. The association with neutropenia was dose-independent, whereas for diarrhea it was restricted to patients receiving medium- or high-dose irinotecan, but not low-dose irinotecan (<125-150 mg m^-2^). The coadministration of 5-FU was not found to affect the association between *UGT1A1* polymorphisms and neutropenia; for diarrhea, the analysis was underpowered for firm conclusions. Importantly, in contrast to irinotecan-induced toxicity, the UGT1A1*28 and UGT1A1*6 polymorphisms showed no association with the objective response rate to irinotecan.

Two other aspects of the PGx of irinotecan/*UGT1A1* merit attention: first is the possibility of increasing the dose of irinotecan above 350 mg m^-2^ in patients with the wild-type genotype, UGT1A1*/*1. Thus, Innocenti et al. 46 reported that the maximum tolerated dose (MTD) of irinotecan was 850 mg, 700 mg and 400 mg in patients with the UGT1A1*/*1, *1/*28 and *28/*28 genotypes, respectively. Accordingly, the standard dose of 350 mg m^-2^ should be reduced by ∼40% in patients with the *28/*28 genotype, but results in underdosing by ∼10% and 34% in patients with the 1*/*1 and *1/*28 genotypes, respectively. Trials exploring the outcome of irinotecan dose adjustment according to *UGT1A1* genotype are underway. A second point that merits consideration is the cost-effectiveness of PGx testing (*UGT1A1* genotyping) for irinotecan. This has been examined in several studies, with ambiguous results 29,30,32. Recently, Roncatto et al. 47 adopted a novel strategy to address this issue based on the cost of managing irinotecan-induced toxicity. For Italian colorectal cancer patients, the estimated costs were 6-fold greater for the UGT1A1*28/*28 genotype (4,886 euros) than the wild-type genotype UGT1A1*1/*1 (812 euros); for heterozygous patients with the *1/*28 genotype, the estimated costs were 1,119 euros, still significantly higher than for the wild-type patients. The authors argued that the differential of toxicity management cost by *UGT1A1* genotype is a step toward demonstrating the clinical utility of PGx testing.

The DWPG guidelines recommend dosage adjustment only in patients with the UGT1A1*28/*28 genotype who are candidates for therapeutic schemes with high-dose (>250 mg m^-2^) irinotecan. The recommendation is to reduce the initial dose by 30%, followed by dose adjustment in response to neutrophil count. For doses <250 mg, no adjustment is suggested. The CPIC issued no guidelines for the irinotecan/*UGT1A1* pair.

### *CYP2D6* and tamoxifen

**Figure 4:**
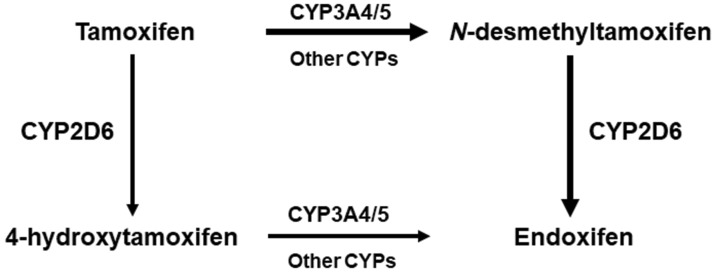
Metabolic routes of tamoxifen leading to the active metabolites endoxifen and 4-hydroxytamoxifen, showing the major CYP enzyme participating in each step.

Tamoxifen, a selective estrogen receptor modulator, is used successfully for long-term adjuvant therapy in breast cancer. Being a prodrug, tamoxifen must be converted into active metabolites, primarily 4-hydroxy tamoxifen and 4-hydroxy N-desmethyltamoxifen (endoxifen), to fully exert its pharmacological actions. Compared with tamoxifen, endoxifen has substantially lower steady-state concentrations in blood but has at least 100-fold higher affinity for the estrogen receptor. The metabolic pathways for tamoxifen in the human liver, summarized in Figure 4, comprise several enzymes of the cytochrome P450 (CYP) family, but CYP2D6 is the rate-limiting step for the formation of endoxifen.

The *CYP2D6* gene is highly polymorphic, with over 80 variants listed in the Human CYP Allele Nomenclature Database (www.pharmvar.org), many of which affect the gene product, resulting in wide interindividual differences in CYP2D6 activity. Four major CYP2D6 metabolic phenotypes are recognized, namely, ultrarapid (UM), normal (NM), intermediate (IM) and poor (PM), of which UM corresponds to the highest activity and PM to the lowest. CYP2D6 phenotypes may be identified using “phenotypic probes” (i.e., drugs that are selectively metabolized by CYP2D6, such as dextromethorphan and metoprolol) or inferred from the *CYP2D6* genotypes using an activity scoring system 48. For example, carriers of two null *CYP2D6* alleles (e.g., CYP2D6*4/*5) are inferred to be PMs, individuals with no defective alleles are NMs (CYP2D6*1/*1), and those carrying multiple copies of functional alleles (e.g., CYP2D6*1x4/*2) are EMs. 

There is consistent evidence that patients carrying decreased- or no-function CYP2D6 alleles show lower plasma endoxifen concentrations than those having the homozygous normal genotype. Interestingly, doubling the tamoxifen daily dose (20 to 40 mg) eliminated the differences in endoxifen concentration during tamoxifen treatment in IM but not PM patients 49. These findings support the notion that *CYP2D6* phenotype is a strong predictor of endoxifen concentration and suggest that increasing the tamoxifen dose may be a strategy to maintain an effective plasma endoxifen concentration in patients carrying decreased-function or null *CYP2D6* alleles 49,50. However, it is still unclear whether endoxifen (or the ensemble of active tamoxifen metabolites) concentration is associated with treatment efficacy. There are several excellent reviews of this topic, and some are referenced here 50-52. Two very recent systematic analyses of data from more than 13,000 patients reported “no clinically important association between *CYP2D6* genotype and breast cancer survival in tamoxifen-treated women” 51 and that the overall effects of different CYP2D6 phenotypes on breast cancer outcomes are not clear 52. Accordingly, professional societies, such as the ASCO or National Comprehensive Cancer Network (NCCN) do not recommend PGx testing for *CYP2D6* prior to tamoxifen therapy. However, the DPWG has published guidelines with recommendations to “...consider aromatase inhibitors for postmenopausal women” and “avoid the coadministration of CYP2D6 inhibitors” in CYP2D6 PM and IM patients. Recently, the CPIC has also published guidelines focusing on the role of CYP2D6 genotype in the adjuvant treatment of estrogen receptor-positive breast cancer 53. Alternative hormonal therapy (aromatase inhibitors) is recommended for CYP2D6 PM patients and has been proposed as a possibility to be considered for IM patients. Standard doses of tamoxifen are recommended for patients with the NM, IM or UM phenotypes. The coadministration of CYP2D6 inhibitors is to be avoided in all except CYP2D6 PM patients. Regarding regulatory agencies (Table 2), the FDA included PGx information on the tamoxifen label but made no recommendations regarding PGx testing, whereas testing is recommended by the PMDA (Japan) and required by the HCSC (Canada).

### PGx in Brazil

The Brazilian population, currently in excess of 210 million individuals, is highly heterogeneous and admixed, a fact that has far-reaching consequences for PGx 54-56. Recognition of this fact prompted the creation of a nation-wide network, the Rede Nacional de Farmacogenética, or Refargen 57, which presently comprises 19 research groups distributed over four geographical regions of Brazil (www.refargen.org.br). The Refargen website presents data on the frequency of PGx polymorphisms in the Brazilian population obtained from two studies: the first study enrolled 1,034 healthy individuals from four geographical regions who were genotyped for 44 SNPs in 16 genes; the second study included 270 healthy subjects from the Southeast region who were genotyped for over 1,900 polymorphisms in 215 pharmacogenes. All participants in both Refargen studies were also genotyped with panels of ancestry-informative markers, which provided data for assessment of the influence of the individual proportions of Native American (Amerindian), European and African ancestry on the distribution of the PGx polymorphisms 56,58,59. Collectively, these studies reveal the following PGx implications: (I) The distribution of PGx polymorphisms among Brazilians varies across geographical regions and self-reported “race/color” categories and is best modeled as continuous functions of individual proportions of European and African ancestry. (II) The differential frequency of polymorphisms impacts the calculations of sample sizes required for adequate statistical power in clinical trials performed in different strata of the Brazilian population. (III) Extrapolation of PGx data from well-defined ethnic groups to Brazilians is plagued with uncertainty. As a corollary to these conclusions, PGx studies in Brazilian cohorts should be encouraged to generate supporting data for the implementation of PGx-informed prescription in our population.

### PGx studies of antineoplastic drugs in Brazilians

This section highlights selected examples of Brazilian studies related to the PGx of the drug-gene pairs listed in the CPIC and/or DWPG guidelines for cancer chemotherapy agents. Table 2 presents data from the Refargen website and other sources for the frequency, among Brazilians, of the pharmacogenetic variants listed in these guidelines.

### *TPMT* and thiopurines

To the best of my knowledge, complemented by a PubMed search using the terms “Brazil* AND TPMT,” PGx testing for TPMT has not been used routinely to guide thiopurine therapy in Brazil. However, this search revealed a number of studies assessing the frequency of *TPMT* polymorphisms and the distribution of phenotypes among Brazilians. The combined frequency of the TPMT*2, *3A and *3C nonfunctional alleles was 4.5% in the overall Refargen cohort (n=1034 healthy individuals), with relatively similar distributions in self-reported White, Brown (i.e., *Pardo*) and Black subjects (Table 2). These data are consistent with those of previously published studies in Brazilian healthy subjects 60,61 and children with ALL 62. Reis et al. 60 quantified the TPMT enzymatic activity in 306 Brazilians and observed a trimodal distribution of high (89.9% of individuals), intermediate (9.8%) and low (0.3%) TPMT metabolizer phenotypes; these proportions are in excellent agreement with the results of the pivotal study performed by Weinshilboum and Sladek 63.

Collectively, the available data for Brazilians indicate that 0.3% of the population is at a 100% risk of severe toxicity when exposed to the standard doses of thiopurines and that ∼10% are at an increased risk of toxicity with such doses. It is likely that these numbers, especially the very low prevalence of the homozygous mutant *TPMT* genotype with greatly reduced TPMT activity, contribute to the nonadoption of PGx testing for thiopurine in Brazil. Nevertheless, it must be emphasized that preemptive TPMT genotyping is a relatively simple laboratory procedure, which may identify patients at a 100% risk of severe, potentially fatal hematological toxicity.

### *DPYD* and fluoropyrimidines

Of the four variants listed in the CPIC and DPWG guidelines, the DPYD*2A and *13 alleles were not detected in the Refargen cohort of 270 healthy subjects, while the other two (2646A>T and HapB3) were not investigated in this cohort (Table 2) However, Cunha-Junior et al. 64 identified three heterozygous carriers of the deleterious mutations DYPD*2A (n=1) and 2846A>T (n=2) among 33 gastrointestinal patients treated with 5-FU. These three patients developed severe, grade 3-4 toxicity, whereas no deleterious mutations were detected among patients with grade 0-1 toxicity. This revealed a 100% specificity and 23% specificity for *DYPD* genotyping in predicting 5-FU-induced severe toxicity. These values are in good agreement with those of international studies (see above). Cunha-Junior et al. 64 also explored the use of ^13^C-uracyl breath tests to predict 5-FU toxicity and concluded that it has moderate accuracy in discriminating patients susceptible to severe 5-FU toxicity *versus* mild or no toxicity.

Gallarza et al. 65 compared *DPYD* genotyping *versus* phenotypic methods (ratio of uracil (U) to dihydrouracil (UH_2_) concentration in plasma and saliva) as predictors of severe fluoropyrimidine toxicity in 60 patients with gastrointestinal tumors. Grade 3-4 toxicity was observed in 21 patients (35%). The deleterious variants investigated, namely, DPYD*2A and *13 and the SNP *Y186C* (rs115232898), were not detected in the study cohort, while the UH_2_/U metabolic ratios showed moderate correlations (r_s_=-0.282 in plasma and -0.515 in saliva) with toxicity grade. The authors suggested that these ratios, especially in saliva, may be promising functional metrics for assessing the potential for fluoropyrimidine toxicity.

### *UGT1A1* and irinotecan

Frequency data for the UGT1A1*28 allele in Brazilians was first reported by Fertrin et al. 66. Our group 67,68 extended this analysis to UGT1A1*36 and *37 (Table 2). Collectively, the two sets of data indicate that the UGT1A1*28 allele is quite common (32-40%) among Brazilians, whereas the alleles *36 and *37 are rare (0.4-1.1%). A PubMed search using the terms “Brazil*, irinotecan, UGT1A1” disclosed no entries in the database. However, an extended search revealed that Hahn et al. 69 developed and validated an analytical method for the quantification of irinotecan and its active metabolite, SN-38, in dried blood samples, which might prove suitable for clinical use.

### *CYP2D6* and tamoxifen

Different aspects of the PGx of tamoxifen have been explored in a number of original studies in Brazilians, and Vianna-Jorge et al. 70 published a careful overview of the impact of functional polymorphisms in metabolizing enzymes on the treatment of breast cancer. I will briefly comment on selected results from the original studies.

Friedrich et al. 71 reported the distribution of *CYP2D6* allele variants, genotypes and inferred metabolic phenotypes in a cohort of 1,034 healthy Brazilians self-reported as White, Brown or Black and recruited in four different geographical regions (North, Northeast, Southeast and South) of Brazil. The overall data for metabolic phenotypes are summarized in Table 2. The most frequent phenotype in the entire cohort was EM (83.5%); PM and UM accounted for 2.5% and 3.7%, respectively. Similar frequencies for inferred CYP2D6 phenotypes in breast cancer patients were reported in a series of articles by Antunes and colleagues 72-75. These authors also measured the plasma levels of tamoxifen and metabolites and investigated associations between the plasma concentrations per se or expressed as concentration ratios, with CYP2D6 phenotypes inferred from genotypes or obtained using the phenotypic probe dextromethorphan. Among their many interesting observations was the conclusion that “CYP2D6 genotyping and/or phenotyping could not fully predict endoxifen concentrations” in plasma. Antunes et al. 75 also explored the influence of the CYP3A4*22 defective variant and CYP3A4 phenotype derived from the (omeprazole)/(omeprazole sulfone) plasma concentration ratio on tamoxifen metabolism in breast cancer patients. Their results confirmed the contribution of CYP3A4 to the bioactivation of tamoxifen and revealed that this contribution “becomes increasingly important in cases of reduced or absent CYP2D6 activity.”

Two other studies examined the frequency of selected *CYP2D6* alleles on recurrence 76 and disease-free survival 77 in Brazilian women with breast cancer. Both studies included relatively small cohorts (n=80 and 58), genotyped limited numbers of known functional *CYP2D6* variants and did not examine copy number variation, which collectively influenced the conclusions drawn.

### Final considerations

PGx seeks to understand the genetic profile of the individual patient to optimize drug therapy, increase efficacy, prevent/reduce adverse effects and improve cost-effectivness. Pharmacogeneticists fully realize that the genetic component is one of several variables that modulate drug response. Thus, it is intuitive that (I) the larger the relative contribution of genetic factors to the pharmacological response, the greater will be the importance of PGx-informed drug prescription and (II) the more polygenic is a drug response, the more complex will be the clinical implementation of PGx. Accordingly, PGx has been most successful when dealing with monogenic or oligogenic drug traits; distinct examples are the association of *HLA* haplotypes with the toxicity of abacavir and carbamazepine.

Polymorphisms in single genes are also thought to be key determinants of the toxicity (thiopurines, 5-FU and irinotecan) or efficacy (tamoxifen) of the antineoplastic drugs examined in this review. Nevertheless, the PGx-informed prescription of these drugs is, to the best of my knowledge, rarely or never performed in most clinical settings in Brazil. This situation reflects the discouraging impact of the aforementioned barriers to the routine clinical implementation of PGx tests. Overcoming these barriers requires collaborative efforts at several levels, including (I) the availability of PGx tests at an affordable cost and in a timely manner; (II) the standardization of PGx testing and result reporting; (III) educational opportunities to improve the understanding of which test to order, when to order tests and how to interpret the results; (IV) access to EMR systems providing CDS and to guidelines for PGx-informed prescription; and (V) evidence of clinical utility and cost-effectiveness applicable to the pertinent clinical setting. PGx testing has the potential to greatly benefit patients by enabling precision medicine applied to drug therapy, ensuring better efficacy and reducing the risk of adverse effects. Refargen, the Brazilian Pharmacogenetics Network (www.refargen.org.br), provides a framework for these collaborative efforts and welcomes professionals and students interested in contributing to the expansion of PGx investigation and clinical adoption Brazil.

## ACKNOWLEDGMENTS

Research in GS-K's laboratory is supported by grants from the CNPq, Faperj and DECIT/Ministry of Health, Brazil.

## Figures and Tables

**Table 1:** Guidelines and label information for germline variants in pharmacogenes associated with antineoplastic drugs.

Drug	Gene	Guidelines^a^		Label information^b^			
		CPIC	DPWG	FDA	EMA	PMDA	HCSC
Azathioprine	*TPMT*	+	+	Testing recommended		Actionable PGx	Actionable PGx
Mercaptopurine	*TPMT*	+	+	Testing recommended	Actionable PGx		Actionable PGx
5-Fluorouracil	*DPYD*	+	+	Actionable PGx		Actionable PGx	
Capecitabine	*DPYD*						
Irinotecan	*UGT1A1*		+	Dosing information		Testing recommended	Actionable PGx
Tamoxifen	*CYP2D6*	+	+	Informative PGx			Testing required

^a^CPIC, Clinical Pharmacogenetics Implementation Consortium (https://cpicPGx.org/guidelines); DPWG, Dutch Pharmacogenetics Working Group (https://www.pharmgkb.org.page.dpwg).

^b^Source: PharmGKB website, https://www.pharmgkb.org/view/drug-labels.do. FDA, United States Food and Drug Administration; EMA, European Medicines Agency; PMDA, Pharmaceutical and Medical Devices Agency (Japan); HCSC, Heal Canada/Santé Canada.

**Table 2:** Frequency (%) among Brazilians of polymorphisms in pharmacogenes listed in the CPIC and DPWG guidelines for antineoplastic drugs.

Gene/*allele	ID number (variant)	Self-reported race/color^a^				Sample	Reference
		White	Brown	Black	Total	size^b^	
*TPMT*						1034	Refargen website
*2	rs1800462 (c.238G>C)	0.4	1.4	1.4	0.9		
*3A	rs1800460 + rs1142345	0.9	1.1	1.0	1.0		
*3B	rs1800460 (c.460G>A)	0	0	0	0		
*3C	rs1142345 (c.719A>G)	1.7	3.7	1.7	2.6		
							
*UGT1A1*						268	Santoro et al. (68)
*28	rs8175347 (7 TATA repeats)				35.2		
*36	rs8175347 (5 TATA repeats)				1.1		
*37	rs8175347 (8 TATA repeats)				0.4		
							
*DPYD*						270	Refargen website
*2A	rs391829 (c.1905+1G>A)	0	0	0	0		
*13	rs55886062 (c.1679T>G)	0	0	0	0		
							
CYP2D6 phenotype^c^	Activity Score^d^					1030	Friedrich et al. (71)
UM	>2	1.2-4.6	1.2-4.6	0-4.7			
IM	0.5-1	3.4-6.9	0-12.7	8.2-12.9			
PM	0	2.3-6.7	0-3.4	1.2-6.2			

^a^According to the race/color categories adopted by the Brazilian Census, Brown corresponding to "Pardo."

^b^Number of individuals.

^c^Metabolic phenotypes inferred from the *CYP2D6* diplotypes: UM, ultrarapid metabolizer; IM, intermediate metabolizer; PM, poor metabolizer.

^d^Activity Score, as decribed by Gaedigk et al. (48).
